# Hidden motion made known – rotational X-ray tracking reveals spinning colloids

**DOI:** 10.1107/S2052252514008549

**Published:** 2014-04-30

**Authors:** Alec Sandy

**Affiliations:** a X-ray Science Division, Argonne National Laboratory, 9700 S. Cass Ave., Lemont, IL 60439-4857, USA

**Keywords:** rotational X-ray tracking, rotational dynamics, colloidal gels

## Abstract

Rotational x-ray tracking (RXT) is demonstrated as a promising new tool for investigating previously unobservable motion in crystalline nanoscale colloids. Its potential utility is demonstrated by applying it to measurements of the local viscoelastic properties of a gel–colloid nanocomposite.

Over the past two decades or so, there has been increasing interest and development in measuring slow dynamics in disordered systems at the nanoscale. Technologically, this interest stems from a desire to understand the stability and rheological behavior of emulsions and colloidal suspensions – materials of particular relevance to the food and consumer products industries, for example (Dave *et al.*, 2007 ▶). More fundamentally, however, stability and nanoscale motion in these types of materials touch upon larger problems in science such as the nature of dynamics in glassy materials and the physics of jamming (Liu & Nagel, 1998 ▶). Some of the techniques that have been developed over recent years to study the dynamic properties of these materials include X-ray photon correlation spectroscopy (XPCS) (Dierker *et al.*, 1998 ▶) and speckle visibility spectroscopy (SVS) (Dixon & Durian, 2003 ▶). Both of these techniques, however, when performed with X-rays that are particularly sensitive to motion at the nanoscale require stable and very bright coherent X-ray beams that limit their implementation to only large specialized X-ray facilities at select locations in the world. Another important limitation, and this also applies to direct imaging microscopy techniques performed with visible light on analogous systems at larger length scales, is that these measurements are only sensitive to changes in the exact spatial arrangement of the constituent particles and, unless pathologically novel particles are incorporated into the material under investigation, are not sensitive to the orientation or rotation of the particles. In many examples, however, because the materials being studied are at limits of very small or very hindered motions, this requirement limits the applicability of these techniques. In fact, measurements of the angular fluctuations of the constituent particles, not possible by XPCS or SVS, are the only way to measure the local viscous forces in glassy materials but hitherto have not been possible or have only been demonstrated in extremely limited cases (Shinohara *et al.*, 2013 ▶).

To address these shortfalls, Liang and collaborators have developed the new technique of rotational X-ray tracking (RXT) and demonstrated its power by applying it to a study of small crystalline particles nominally immobilized by the fact that they form a colloidal gel under appropriate conditions (Liang *et al.*, 2014 ▶). A gel is an extended disordered network with jelly like structural properties. As such, one might think of the gel in its unperturbed state as being immobile. As demonstrated, however, by RXT, the ostensibly static colloidal particles in the colloidal gel actually undergo angular motion and the precise nature of observed rotational motion is a sensitive and unique measure of the nanoscale elastic properties of the gel network.

The RXT technique requires a relatively high flux X-ray beam, though not a coherent X-ray beam, and crystalline colloidal particles that exhibit Bragg diffraction. The size of the particles can vary depending on the nature of the X-ray source and the volume fraction of the particles but for the studies reported by Liang *et al.*, 340 nm alumina particles at a volume fraction of 40–50% were studied in decanoic acid, a so-called fatty acid, as a function of temperature. When the X-ray beam from a synchrotron X-ray source illuminates the sample and a sensitive area detector is placed at the Bragg condition for reflection from a particular set of crystal planes, then an ‘instantaneous’ snapshot of the scattered X-ray beam reveals the Bragg diffraction spots from each particle that is oriented such that the scattered beam illuminates the detector (typically a very small number of the particles, five in the reported study, because suitable detectors only collect a small fraction of the Debye–Scherrer powder diffraction ring). The actual rotational tracking is done by collecting and following the small number of diffraction spots that are observed to move in the detector even when the sample is structurally static in the gel phase (completely static samples like dried colloidal particle powders do not show motion). RXT is sensitive to the two types of rotation shown in Fig. 1 ▶ but not sensitive to rotation about the normal to the Bragg planes. Nevertheless, despite this limitation, Liang *et al.* argue that the technique is sensitive to angular motions as small as 8 µrad in time steps as small as 1 ms.

As a specific example of this new technique, Liang *et al.* present RXT measurements of crystalline alumina colloids dispersed in a fatty acid as the temperature of the fatty acid is raised from the ‘solid’ state to the liquid state. As shown in Fig. 2 ▶, at lower temperatures in the fatty solid phase, unidirectional drift is observed while exactly at the transition into the liquid/gel phase anomalous behavior such as convective motion is observed. Finally, in the phase where the alumina particles form a gel network, stochastic behavior is observed. The stochastic motion in the gel phase is analyzed quantitatively in their paper and sub-diffusive rotational motion of the colloidal particles is observed. The authors further extend this analysis and show how unique information about the microscopic elastic properties of the gel can be determined from their detailed measurements.

In summary, the work by Liang *et al.* presents a comprehensive explanation of the newly created technique of rotational X-ray tracking that demonstrates how careful measurements using relatively intense X-ray beams and sensitive detectors can be used to measure otherwise unobtainable dynamical properties of technologically and scientifically relevant novel materials.

## Figures and Tables

**Figure 1:**
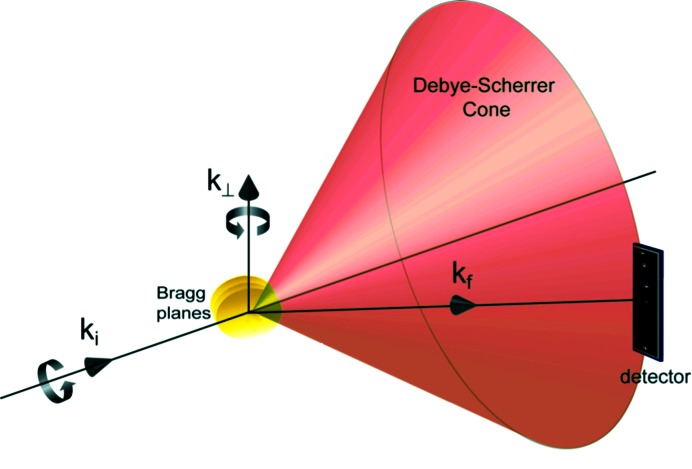
The Bragg scattering geometry of the experiment (adapted from Liang *et al.*, 2014 ▶).

**Figure 2:**
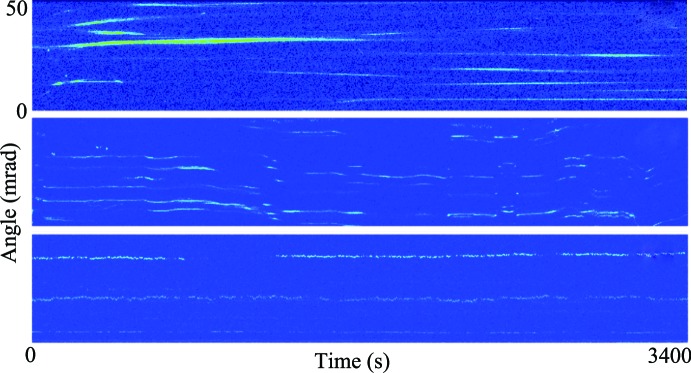
Rotational behaviors of alumina nanocrystals at various temperatures (adapted from Liang *et al.*, 2014 ▶). Top, −25.5°C, below the melting point of decanoic acid; middle, 30.6°C, the melting point; and bottom, slightly above the melting point.
